# No Evidence of Transfer of Learning Between Problem-Solving Tasks Using Different Transformation Rules

**DOI:** 10.1162/OPMI.a.338

**Published:** 2026-03-15

**Authors:** George Gabriel, Faisal Mushtaq

**Affiliations:** School of Psychology, University of Leeds, Leeds, UK; NIHR Leeds Biomedical Research Centre, Leeds, UK

**Keywords:** problem solving, skill learning, string transformation, longitudinal study, working memory

## Abstract

As demand for problem-solving skills continues to grow, so does the need for efficient training methods. A potential method of achieving efficient training of problem-solving skills is to design a training programme that maximises transfer of learning between problem domains. However, current models of problem-solving make contrasting predictions about the conditions under which cross-domain transfer is possible. While pattern-recognition models using state-action associations allow only limited transfer to tasks with different actions, heuristic search-based models using state-value estimates can reuse learning across tasks, potentially achieving greater transfer. In this study, we consider the case of transformation problem-solving tasks where each task uses a distinct and non-overlapping set of transformation rules. Participants trained using tasks from one taskset over four consecutive days and completed pre- and post-training probes on the untrained set. Training improved participants’ solution rate, efficiency, and decision speed, but there were no reliable improvements for the untrained tasks beyond what could be explained by direct practice during the probe blocks. There were no consistent trends in participants’ self-reported strategies across sessions, but use of a strategy involving explicit deliberation over which actions to take was consistently associated with better decisions. We note that the absence of transfer contrasts with the predictions of the heuristic search account of problem-solving in which a learned rule-independent state-value heuristic is reused across tasksets to decide actions. We discuss potential reasons for this absence of transfer and its possible implications for future models of problem-solving and for the training of problem-solving skills.

## INTRODUCTION

Problem-solving is an essential component of human cognition. From abstract skills like algebra to everyday tasks like budgeting and time management, problem-solving abilities are ubiquitous in our personal and professional lives. Recent technological developments including generative AI have led to an increased recognition of the importance of human problem-solving and analytical skills (Mäkelä & Stephany, 2025), reigniting long-standing calls for improved training in these areas (Funke et al., 2018; Jonassen, 2010; Rossi & Dickerson, 2024). However, the same breadth of application areas that makes problem-solving skills useful also raises questions about how to efficiently train them.

Training multiple task-specific skills may be practically challenging due to time and resource constraints, as shown by recent difficulties integrating computing classes into already crowded school curricula (Almdahem, 2024; Bruno et al., 2022; Ofsted, 2022). A more efficient general-purpose approach to training problem-solving may therefore be appealing; but it is unclear whether problem-solving proficiency can be achieved across multiple domains through non-specific training.

Evidence from the cognitive training literature has shown that practicing specialised cognitive abilities rarely results in performance gains beyond the trained tasks (Simons et al., 2016; von Bastian et al., 2022). In the present paper, we assess whether the same may be true of problem-solving skills.

We focus on a broad category of problem-solving tasks classically termed “transformation problems” (Greeno, 1978). These problems require the solver to apply transformation rules to transform a task state into a goal state, possible in multiple steps. This structure encapsulates many educationally relevant problem-solving tasks including algebra, geometry, and aspects of computing such as software debugging.

The computational study of transformation problems has been pursued since at least the 1950s (Moore & Anderson, 1954; Newell et al., 1958; Newell & Simon, 1972), leading to several important theoretical results. It has long been recognised that novice behaviour on transformation problems differs substantially from that of domain experts, with a transition towards less deliberative or working memory-dependent processes with increasing expertise (Gick, 1986; Gobet, 1997; Shanteau, 1992; Sweller et al., 1983). While a detailed review of current models of problem-solving is beyond the scope of this paper, it is informative for our purposes to distinguish two categories of putative computational mechanisms thought to underpin problem-solving in a broad subset of these models. We refer to these mechanisms as search-based or pattern-recognition and association-based.

Models emphasising heuristic search select transformation rules based on the quality of the states that would result from using those rules (Gigerenzer & Gaissmaier, 2011; Salhi, 2017). The problem-solver internally computes an approximation of the state resulting from the use of a given rule or rule sequence in the current state. An assessment of the value or utility of the resulting state is then made using a function called a heuristic (Callaway et al., 2022; Huys et al., 2015; Krusche et al., 2018; Newell & Simon, 1972; van Opheusden et al., 2023). The estimated value may incorporate information about the long-term consequences of an action by searching multiple steps ahead, and may allow comparison between multiple possible rules by evaluating each of their resulting states. The computed value estimates are then used to decide which rule or rule sequence to apply in the current state.

A second class of models simultaneously emphasizes pattern recognition and the acquisition of reusable long-term memory representations. The problem-solver learns to recognise patterns in the current state (Bilalić et al., 2010; Chase & Simon, 1973; Shanteau, 1992; Sheridan & Reingold, 2017) and associates them with rule sequences or more abstract “templates” stored in long-term memory (Gobet, 1997; Gobet & Simon, 1996; Tenison & Anderson, 2016). When a task state is encountered, features of that state are automatically extracted and used to trigger associative recall of an appropriate rule sequence or template. The precise mechanisms of association and the nature of the stored representations vary between models; but in each case learning proceeds by improving the recognition of task-relevant patterns, increasing the availability of useful structures in long-term memory, or enhancing the quality or strength of associations between the two.

Each of the above-described mechanisms can allow transfer of learning between tasks which share the same rules (Cooper & Sweller, 1987; Sweller et al., 1982), or tasks whose rules can be mapped onto each other (Gentner, 1983; Gick & Holyoak, 1980). Cross-task reuse of learning is an essential component of how domain-specific expertise is achieved. Practicing a finite set of tasks using a given ruleset leads to improved performance on unpractised tasks which use the same ruleset. However, when transformation rules are not shared between tasks, the possibility of learning from one task being reusable in the other is less clear.

Problem-solving algorithms in which learning is independent of the specific transformation rules are more likely to support generalisation across different rulesets. Of the pattern-recognition and search-based mechanisms described above, only the latter can achieve this. While pattern-recognition depends upon an association between task state and specific rules or rule sequences, search using a state-value heuristic does not. As the state-value heuristic can be computed from only a task state and goal state, the value of any task state can be estimated independently of the rule or rules which led to that state. The same state-value heuristic could therefore be used to assess the value of states arising from any transformation rules, regardless of whether they were available during training. The extent to which transfer is possible between tasks using different rulesets then depends upon how well the learned state-value heuristic for the trained tasks approximates the true state-value for the untrained tasks. This observation allows us to empirically assess whether the state-value heuristic is likely to play an important role in problem-solving performance.

The viability of transfer in the absence of shared transformation rules also has important implications for the training of problem-solving skills. If transfer of learning is limited to tasks sharing the same or similar rules, then achieving general proficiency in multiple task domains may require targeted training in each of those domains. Conversely, if transfer can arise between tasks not sharing the same rules, then training a subset of tasks may be sufficient to achieve proficiency in multiple domains, potentially allowing for the development of more efficient general-purpose training.

In this study, we developed two problem-solving tasksets which used different and non-isomorphic rules, but otherwise used identical task presentation. Participants trained on one taskset for four consecutive days and completed an initial and post-training probe block comprising tasks from the untrained taskset. By comparing performance gains and changes in behaviour on the trained and untrained task domains, we assessed the extent and possible mechanisms of transfer between the two domains.

We observed improvements in task solution probability on the trained tasks, and smaller improvements on the probe tasks, consistent with learning during probe trials. The efficiency of participants’ solutions improved with training, and we observed limited improvements in efficiency for the probe condition. Further analyses of participants’ self-reported strategies found no consistent changes in problem-solving strategy across sessions, though use of a strategy involving deliberation over which actions to take was consistently associated with better action choices. Our results suggest that transfer between problem-solving tasks using different transformation rules may be limited. We discuss the implications of these findings for training of problem-solving skills and the development of future models of problem-solving.

## METHODS

### Participants

Seventy-three participants were recruited using the Prolific experiment platform and were randomly assigned to one of two participant groups at the beginning of their first session. Participants were eligible to participate in the study if their Prolific accounts reported that they were between 22 and 40 years of age, their first and primary languages were both English, and they were both born and currently lived in the United Kingdom. Of these participants, 56 qualified for inclusion in our analyses (exclusion criteria described below). Thirty participants were included from Group A (19 female, group mean age 33.3 years, *SD* = 4.27 years), and 26 from Group B (14 female, group mean age 31.4, *SD* = 4.44 years).

All sessions were completed remotely via a web-based system by participants using their own devices. Only desktop computers and laptops running Microsoft Windows or MacOS were allowed. Participants were paid £5 per completed session of the study, with payments made immediately upon completion of the session. All participants gave informed consent via an online survey before beginning the first session. The study was approved by the ethical review board of the University of Leeds School of Psychology.

### Experiment

In each session, participants completed two tasks via a web-based system: the problem-solving task and an additional working memory task.

#### Problem-Solving Task.

The problem-solving task used in this study was a generalisation of classic transformation tasks based on rearranging logic expressions (Greeno, 1978; Moore & Anderson, 1954). For each problem, participants were presented with a string of symbols comprising the current state of the task, and a second string comprising the task goal. Participants were also shown a list of transformation rules comprising two strings of consecutive symbols separated by a rightward-pointing arrow. The rules could be used to transform subsections of the current state by replacing substrings matching the left-hand side of the rule with the substring on the right-hand side of the rule.

**Figure 1.**
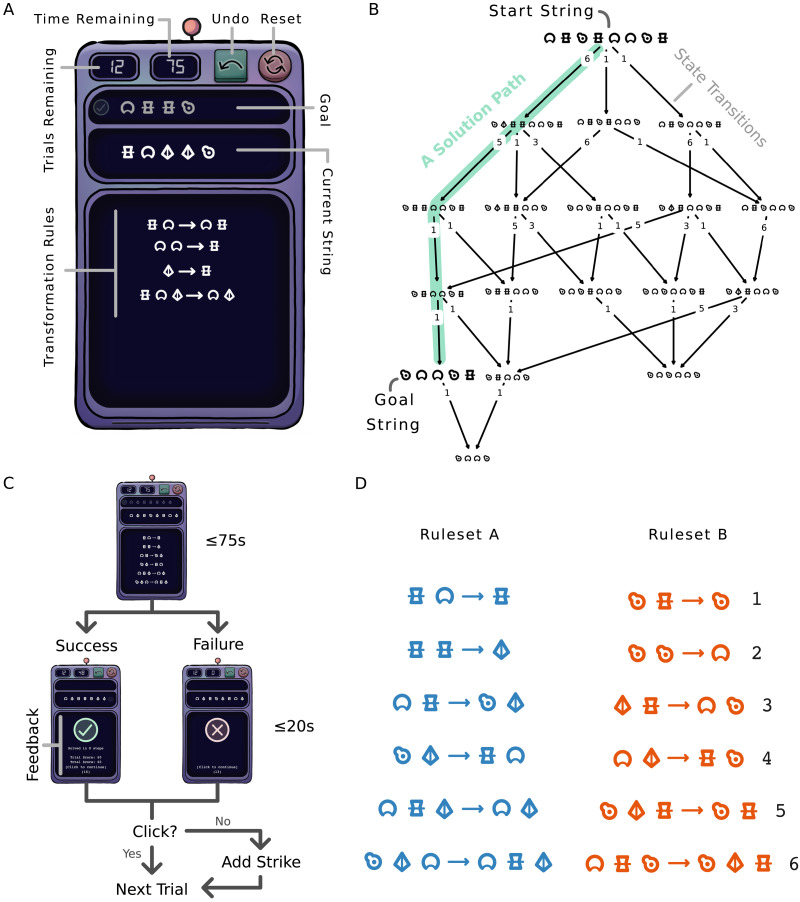
Problem-solving task components. (A) The graphical user interface for the problem-solving task. The indicated transformation rules were not used in the actual study. (B) An example state graph for a task from taskset B, showing all possible states reachable from the given start state by using ruleset B. Each edge represents an application of a transformation rule (numbered as in subfigure D, ruleset B). The green path is a possible solution transitioning from the start state to the goal state. This task was used in taskset B, and has one of the smallest state spaces of any used task. (C) The flow of task states in a trial of the problem-solving task. Participants have up to 75 seconds to complete the task. If they succeed, they receive feedback on the number of steps they took and a per-trial and cumulative score. If they fail, they are not shown feedback. In both cases, they must click within 20 seconds to proceed to the next trial. Otherwise, a penalty strike is incurred. (D) The two rulesets used to define the actual tasksets used in the problem-solving trials of the study.

Participants could select a rule by clicking it with a cursor. They could also select a position in the current state string by clicking the symbol in that position. When a rule and a position in the current state were both selected (in either order), the software attempted to automatically apply the rule. If the left-hand side of the active rule matched the equal-length substring of the current state starting from the active position, that substring was replaced by the symbols on the right-hand side of the active rule, and the rule and active position were deactivated. If they did not match, the rule and active position were deactivated without changing the current state string, and a short shaking animation played to indicate an invalid attempted rule application. The graphical user interface for the task is shown in Figure 1A.

Participants were instructed to use the presented transformation rules to transform the initial state string such that it was identical to the goal string. Rules could be applied in any order and there was no maximum or minimum number of times each rule could be used (but see the Automatic Abuse Detection methods outlined below). Participants could also undo their moves one at a time by clicking an undo button, or reset the task to its original state using a reset button. A solution to a transformation task is any sequence of rule applications which results in the start state being transformed into the goal state. An example solution for a simple task is shown in Figure 1B.

Two different sets of tasks were generated before the sessions. These tasksets each contained seventy-two different tasks, where the same start state and goal state was never used by more than one task. The tasksets were generated using two non-overlapping sets of transformation rules, with five rules in each set. The structures of the rules (i.e., the number of symbols on the left and right side) were distributed identically in the two tasksets, but the rulesets were designed not to be isomorphic in either inputs or outputs (i.e., one ruleset could not be recreated by substituting each symbol in the other ruleset by a different symbol). The symbols and rulesets used in the problem-solving trials are shown in Figure 1D.

Tasks were selected to meet specific difficulty criteria, based on initial pilot analysis of factors influencing performance on a different set of tasks. Specifically, all tasks had between two and four distinct (but possibly overlapping) solution sequences, required between four and six steps to reach the goal state, and had goal states with length of at least three symbols. Tasks were also chosen such that every state on a solution path had at least one valid next state which was not on a solution path, allowing participants to take “wrong turns” at any point along a solution path. We also removed tasks containing commutative transitions, where a state could be reached by applying a sequence of two rules in either order.

The two sets of seventy-two tasks contained equal numbers of trials with minimum solution path lengths of four, five, and six steps.

During problem-solving trials, we recorded the position and timestamp of each click event on the screen, the timestamp of all activation and deactivation events for rules and state symbols, and the current state string after each transformation.

#### Working Memory Task.

The working memory task was a version of the running digit span task (Bunting et al., 2006). In our implementation, participants were shown a fixation cross on a black screen for 1.5 seconds before a rapid sequence of digits was shown. The digits were also read aloud by a computer voice, and were each displayed for 250 ms. The sequences contained between twelve and twenty-one symbols (inclusive), and the symbols were the nine numerical digits “1” to “9”. The digits were randomly selected such that the sequences never contained the same digit more than three times, the same digit was never repeated more than once in a window of six digits, and pairs of consecutively shown digits were never numerically consecutive either forward or in reverse.

Participants were instructed to recall as many digits as possible from the end of the string. They typed their responses via the keyboard after the sequence ended. There was no time limit for responses.

### Exclusion Criteria

A subset of participants who completed all sessions were excluded from the reported analyses due to improper execution of the problem-solving or working memory tasks. Participants were excluded if they did not solve at least one problem-solving trial in each session, or if they did not correctly recall the most recently shown digit with greater than chance probability in all sessions. Recall probability was computed using binomial regression, as described in the section on Working Memory Capacity. These criteria were intended to remove participants who did not understand the tasks or otherwise did not engage properly with them without removing participants who understood and engaged with the tasks but had lower than average performance. Of the seventy-three participants who completed all sessions, eight were excluded from group A, and nine were excluded from group B.

### Session Structure

Each of the four sessions comprised two blocks of twelve problem-solving trials, followed by a single block of twenty trials of the working memory task.

The first session of the study also included an initial introduction to the task, an interactive tutorial, a task understanding quiz, and a discomfort tolerance survey before the main tasks began. These components were not repeated on subsequent sessions.

#### Discomfort Tolerance Survey.

Participants responded to the eight questions from Factor I of Harrington’s frustration-discomfort scale (Harrington, 2005). They rated their agreement with statements about tolerance for frustrating or uncomfortable tasks on a five-point scale ranging from “absent” to “very strong”.

#### Tutorial and Understanding Quiz.

In the first session, participants also completed an interactive tutorial comprising instructions on the task goal and how to apply the transformation rules. Participants completed one simple practice task solvable in three steps. The rules used in this practice task were not used in either of the non-practice tasksets. Participants were then required to pass an understanding quiz in which they were asked five multiple-choice questions about the functionality of the user interface, including the purpose of the undo and reset buttons, and were required to pick a single rule application that would solve a sample task. Participants answering fewer than four of the five questions correctly were excluded from participating in the study.

#### Problem-Solving Blocks.

The structure of a problem-solving trial is illustrated in Figure 1C. Problem-solving trials each had a maximum completion time of 75 seconds. Trials not solved within this time were recorded as failed, and participants were shown a feedback icon indicating that they failed the trial. When a trial was solved within the time limit, participants were shown feedback comprising the number of rule applications they used to solve the trial and a score for that trial. The score was defined to be ten times the remaining trial time divided by the number of steps taken, rounded down to the nearest integer. Feedback for successful or failed trials was shown for a maximum of 20 seconds, and participants could click to continue to the next trial at any time within this limit. If the participant did not continue within the time limit, they received one penalty strike (see Automatic Abuse Detection).

All blocks of problem-solving trials comprised 12 trials followed by a post-block survey. There were two problem-solving blocks per session. In the first session, the first problem-solving block for participants in Group A was comprised of tasks sampled randomly from taskset B. This was an initial probe block to assess baseline performance on taskset B. For the second block of the first session, both blocks in sessions 2 and 3, and the first block of the final session, participants in Group A completed all tasks in taskset A in random order. These were training blocks to allow participants to become proficient in tasks from taskset A. For the final block of the final session, participants completed randomly selected tasks from taskset B that they had not already completed during session 1. This was a post-training probe block to assess transfer of performance gains to the untrained taskset. For participants in Group B, the tasksets were reversed.

#### Post-Block Surveys.

After each block of the problem-solving task, participants completed a short survey rating their use of different problem-solving strategies and their emotional state during the preceding block.

Three strategies were described to the participants: “work-it-out”, which involved trying to internally evaluate the result of using a rule before applying it; “try-and-see”, which involved applying a rule to see its consequences instead of internally evaluating the result; and “intuition”, which involved following feelings or intuitions about which rule would be good to use without either trying it or working out its consequences in advance.

For each pair of rules, (intuition vs. try-and-see; try-and-see vs. work-it-out; work-it-out vs. intuition) participants gave a percentage score indicating the proportion of trials in the preceding block for which they preferred the first strategy in the pair to the other strategy in the pair.

Participants also provided ratings of their boredom during the block, how enjoyable they found the puzzles in the block, and their frustration during the block, each on a four-point scale from “not at all” to “very much”.

#### Working Memory Block.

Participants completed 20 trials of the working memory task per session, with sequence lengths ranging between 12 and 21 symbols inclusive. Each sequence length was repeated twice in the block, and the order of the trials was randomised. Sequences were independently randomly chosen for all participants and trials and were not repeated across sessions.

#### Automatic Abuse Detection.

As the study was conducted online, it was necessary to include features to discourage inappropriate task behaviours which may have compromised data quality. We used a system of strikes where participants were allowed up to three strikes before being excluded from the current session and all subsequent sessions. Strikes were awarded in the following conditions: The participant: (i) clicked rules or state symbols more than 50 times within a single trial; (ii) navigated to another tab or window while the study webpage was open; or (iii) failed to click to continue within 20 seconds of the post-trial feedback being shown. Participants were informed of these criteria and the possibility of being excluded due to strikes in the information sheet and interactive tutorial.

### Analyses

We computed several measures of performance on the problem-solving and working memory tasks and used regression analyses to identify associations between performance and training.

#### Performance Measures.

##### Proportion of Problems Solved.

Our simplest measures of performance on the problem-solving task relate to the proportion of problems solved. We computed this in two performance measures, one at the block level and one at the trial level.

For the block level performance measure, we computed the proportion of trials in that block that were solved by each participant. We then computed the mean across participants in each group to create a combined performance score for each group and block.

For the trial level performance measure, we computed the proportion of participants solving that trial. As task order was randomised across sessions, participants did not necessarily receive the same tasks for any given trial number. Computing proportion of participants solving each trial grouped by trial number therefore provides an approximation of the overall performance of the participant group which is less sensitive to variations in the difficulty of individual trials.

##### Time Between Rule Uses.

As an approximation of the average time spent deciding which rules to use, we computed the time between successive rule applications. This was achieved by finding sequences of consecutive rule or state symbol selection events (in either order), and then computing the difference in time between the end of one such pair of events and the next pair. We averaged these differences at the trial level for each participant. For block-level analyses we then computed the per-participant means of all trial average times in a block.

##### Action Sequences Efficiency.

We defined a measure of solution efficiency based on the minimum number of successive rule applications required to transition from a given state to the goal state. For each recorded state visited by a participant, we computed the median length of all action sequences transitioning from that start state to the goal state. For states with no path to the solution (without using the undo or reset features), the distance was set to infinity. We then computed the actual number of steps taken by the participant to transition from the given state to the goal, including any attempts at using invalid rules, but ignoring undo and reset events. Finally, to produce a measure of solution efficiency, we grouped the observed states by the corresponding median solution path lengths. For example, all states from which the median solution length was four steps were grouped together for each participant within each block. We then computed the proportion of states in each group from which the observed solution path had length no greater than the group’s median solution path length. Trials which were not solved counted as trials in which the solution length did not meet this criterion.

This process provides, for each participant and each solution path length in each block, a statistic representing the proportion of trials of that length that were solved in no more than the median required number of steps. For example, if a participant has a high value of this statistic for solution distance four in block one, this means they were reaching the goal efficiently in this block when their current state was four steps from the goal.

This measure allows us to distinguish improvements in performance due to increased use of a trial-and-error or brute-force strategy from improvements due to better selection of appropriate rules and rule sequences.

#### Strategy Reports.

We converted the paired-comparison strategy preference percentages into individual weights representing participants’ preferences for each strategy in each block using the following conversion formulae:

$$w_{t}=\frac{xy}{1-x+xy}$$

$$w_{w}=w_{t}\cdot \frac{1-x}{x}$$

$$w_{i}=w_{w}\cdot \frac{1-y}{y}$$

where *w*_*t*_ is the weight assigned to the “try-and-see” strategy, *w*_*w*_ is the weight assigned to the “work-it-out” strategy, and *w*_*i*_ is the weight assigned to the “intuition” strategy. The variables *x*, *y*, and *z* correspond to the participant-reported preferences for the first option in pairwise comparisons between “try-and-see” and “work-it-out”, “work-it-out” and “intuition”, and “intuition” and “try-and-see” respectively. The conversion formulae assume that these values are not equal to 0 or 1, and that the three weights sum to 1. Each weight has a value in the interval [0, 1].

#### Block-Level Regression Analyses.

For our block-level analysis, we used two main types of regression model to identify associations between quantities of interest. To assess associations between training, working memory capacity, use of the three pre-defined strategies, and probability of trial success or action sequence optimality, we used binomial regressions. To assess associations between training, working memory capacity, use of the three pre-defined strategies, and time between actions, we used gamma regression. All regression models were implemented in Python using the ‘statsmodels’ package (version 0.14.4).

The predictor and outcome variables were not standardised before running the regressions, so the beta coefficients can be interpreted in terms of the original units. When block number is used as a predictor, consecutive block numbers from 2 to 7 are used for the training blocks, while the block numbers for the pre-training and post-training blocks are transformed to 0 and 1 respectively to allow easier interpretation of the beta coefficients.

#### Trial-Level Regression Analyses.

We analysed the per-trial probability of trial success with a hierarchical Bayesian logistic regression implemented in Python using PyMC (version 5.26.1). The model included fixed effects for group (A vs. B), trial success (success vs. failure), the probe-versus-training condition, and their interactions. To capture individual differences, the model incorporated participant-level random effects. Each participant had a random intercept, a random slope for cumulative trial number, and a random effect for the probe condition.

We analysed the mean per-trial time between rule uses with a hierarchical Bayesian log-normal regression. As above, the model included fixed effects for group, trial success, the probe-versus-training condition, and their interactions. The model also incorporated participant-level random effects, with each participant having a random intercept, a random slope for cumulative trial number, and a random effect for the probe condition.

The complete specification of each model is described in the Supplementary Methods. Code for the models is included in the data analysis script.

#### State-Value Transfer.

To test whether reuse of state-value heuristics can allow transfer of learning between tasksets, we developed a parametric state-value heuristic and assessed its performance on states sampled from the two tasksets. The purpose of this function is not to model the actual heuristic used by participants in this task (if they do indeed use a heuristic). Instead, it is intended to demonstrate that transfer can, at least in some circumstances, arise from the reuse of a heuristic across tasksets.

The parametric state-value heuristic is defined as follows:

$$v\left(s,g\right)=\text{expit}\left(\beta_{0}+\sum \beta_{i}\cdot f_{i}\left(s,g\right)\right)$$

In this equation, *s* represents the state string to be evaluated, *g* represents the goal state string, *β*_*i*_ are scalar parameters, and *f*_*i*_ are functions mapping state and goal strings to real-valued outputs. In our implementation, there are 13 functions *f*_*i*_ which quantify the presence of certain features in the state and goal strings:

- *f*_1_ measures the proportion of symbols in the current state which match the symbol in the corresponding position of the goal state.
- *f*_2_ computes the difference between the length of the current and goal state strings.
- *f*_3_ computes the difference between the number of unique symbols in the current state and the number of unique symbols in the goal state.
- *f*_4_ computes the length of the longest common subsequence of symbols between the current and goal state strings as a proportion of the length of the goal state string.
- *f*_5_ and *f*_6_ count the lengths of the state string and goal string respectively.
- *f*_7_ and *f*_8_ count the lengths of the longest runs of repeated symbols in the state string and goal string respectively.
- *f*_9_ and *f*_10_ count the lengths of the shortest runs of repeated symbols in the state string and goal string respectively.
- *f*_11_ and *f*_12_ compute the length of the state and goal strings when consecutively repeated symbols have been removed.
- *f*_13_ gives the Levenshtein edit distance between the state and goal strings.

We defined a ground truth state-value for a state and goal pair to be 1 if the goal was reachable from that state (i.e., solvable) and 0 otherwise. Here we operationalise ‘value’ as binary reachability; the aim is to test whether a learned state-evaluation function can generalise across tasksets under minimal assumptions. For each of the two tasksets, we computed all possible states that could arise in those tasks. We then sampled 400 states with ground truth values of 1 and a further 400 states with ground truth values of 0 from each of the two tasksets. These states, together with their corresponding goals, formed the evaluation dataset for our state-value heuristic model.

We used a binomial regression model to predict their ground truth value of task states (i.e., current and goal state) as a function of the feature space representation of those states. To demonstrate that transfer may be possible when using a state-value heuristic, we fitted the model to the above-described data from taskset A and evaluated its performance on taskset B. We also ran the same process with the groups reversed.

To assess the performance of the model, we used 30-fold cross-validation. The model was fitted to the training fold sampled from the training dataset and the accuracy of the fitted model’s predictions were computed on the left-out test data. The generalisation performance of the model was computed using the entire transfer dataset. We computed median, lower quartile, and upper quartile accuracy for the test and generalisation data.

To demonstrate that the performance of the state-value heuristic is reduced when either state or goal information is unavailable, we repeated the above analyses with a subset of predictors containing only goal information (*f*_6_, *f*_8_, *f*_10_, *f*_12_) or only current state information (*f*_5_, *f*_7_, *f*_9_, *f*_11_).

We reason that, if accuracy on both the training and generalisation tasksets is similar, this indicates that transfer using a state-value heuristic is possible. We describe some limitations of this demonstration in the Discussion section.

#### Cursor Movement Speed.

To compute the mean cursor speed within a trial of the problem-solving task, we first identified sequences of rule or symbol activation events corresponding to an attempt at applying a rule. These sequences comprise a rule activation and a symbol activation event in either order. We computed the difference in times between the second and first event in each of these sequences, as well as the difference in position between their corresponding click positions. Using these differences, we computed the average cursor speed in the time period between the clicks. This process was repeated for all click sequences in the trial. For trial-level analyses, the mean of these values was then computed for each participant. For block-level analyses, the mean of all events within a block was calculated for each participant.

#### Working Memory Score.

To quantify participant’s working memory capacity, we used binomial regression to estimate the probability of correctly recalling a digit as a function of its offset from the end of the list. The symbol at offset 0 was the final digit in the string, while the symbol at offset 1 was one symbol before the end, and so on. Working memory scores were computed for each participant and each session individually.

We converted the function mapping offset to recall probability into a single working memory score by finding point along the offset axis at which the recall probability dropped below the chance level. Chance in this case was 1/9, as the state strings were comprised of digits picked from “1” to “9” inclusive. The final working memory score was defined as the offset of the intersection plus one, resulting in a real-valued rather than integral working memory score.

## RESULTS

### Working Memory Capacity

We analysed participants’ performance on the running memory span task to compute a working memory score for each participant. We then used a linear mixed-effects model to examine the effect of session number and counterbalancing group on working memory score, accounting for participant variability as a random effect. This model found a small but statistically significant main effect of session number (*β* = 0.179, 95% CI [0.093, 0.264], *p* < .001), indicating an average increase in working memory score of approximately 0.54 from the first to the final session. The fixed effect of being in counterbalance group B compared to group A was not statistically significant (*β* = 0.010, 95% CI [−0.495, 0.515], *p* < .969). The intercept parameter, corresponding to the mean predicted working memory score in the first session, was 5.590 (95% CI [5.222, 5.957], and the per-participant variance in the random intercept was 0.790. Distributions of participants’ working memory scores across sessions are shown for each group in Supplementary Figure S1.

Given the small but statistically significant change in working memory score across sessions, we control for per-session working memory score in our subsequent regression analyses.

### Average Self-Reported Strategies

To check for trends in participants’ self-reported strategies, we used binomial regressions to independently predict the weighting of each strategy as functions of block number.

For the training blocks, we found no statistically significant association between block number and use of the “try-and-see” (*p* = 0.596), “work-it-out” (*p* = 0.551), or “intuition” (*p* = 0.929) strategies. For the probe blocks, there was also no statistically significant association between use of the “try-and-see” (*p* = 0.098), “work-it-out” (*p* = 0.579), or “intuition” (*p* = 0.304) strategies and the transition from the pre-training and post-training probes.

### Changes in Task Performance

We assessed whether participants’ performance on the training and probe tasksets improved after extended practice of the former. We used three measures of performance to assess different aspects of task behaviour: the proportion of trials solved per block gives an overall measure of task competence; the mean time between rule applications on a block of tasks gives an approximate measure of time spent deciding an action; and the proportion of solutions which were of minimal length gives an indication of the average efficiency of participants’ solutions.

#### Probability of Trial Success Increased More for Trained Than Untrained Conditions.

**Figure 2.**
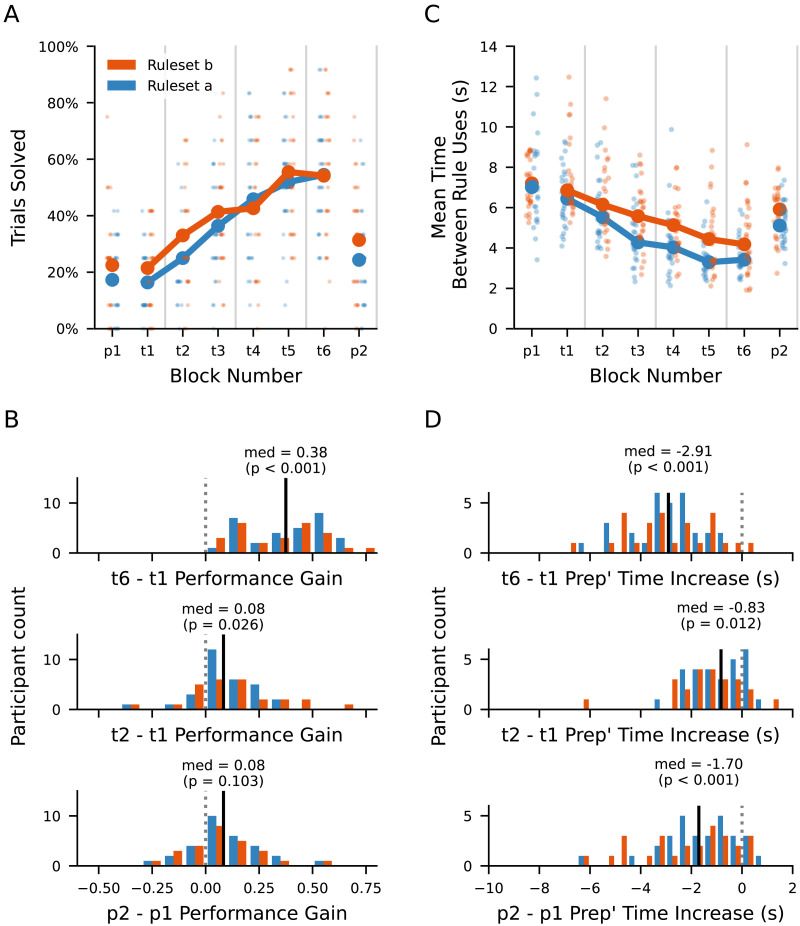
Trends in basic performance measures across probe and training blocks. (A) Grand means (heavy points and lines) of per-participant mean (light points) percentages of trials solved per block, separated by task ruleset. Participants in Group A used tasks with Ruleset A for probe trials (1 and 8), and tasks with Ruleset B for training trials (2 to 7). This was inverted for participants in Group B. Vertical grey lines separate consecutive sessions. (B) Histograms of per-participant difference in proportion of trials solved in indicated pairs of blocks. Black lines and corresponding number labels are medians. Corresponding p-values are from two-sided bootstrap tests for a difference in performance between the two compared blocks, equivalent to a difference of the mean performance change from zero. (C) Medians (heavy points and lines) of per-participant median (light points) time between consecutive rule uses, successful or otherwise. (D) Histograms of per-participant difference in mean time between movements in indicated pairs of blocks.

We computed the mean proportion of trials solved as a function of block number, observing a clear increase for the trained conditions for both participant groups (Figure 2A).

To determine whether performance differed between the two participant groups, we performed two-sided bootstrap hypothesis tests for each of the probe and training blocks. We found no statistically significant differences in proportion of trials solved for participants in group A compared to group B for any block (*p* > .070, in all blocks). Based on this observation, we pooled data across the two groups for our subsequent analyses of performance.

To assess the number of individual participants for whom performance increased, we computed the differences in performance between the last and first training blocks (t6 and t1), the first two training blocks (t2 and t1), and the post-training and pre-training probe blocks (p2 and p1) (Figure 2B). Two-sided bootstrap hypothesis tests found significant increases in the proportion of trials solved from t1 to t6 (*p* < .001) but not p1 to p2 (*p* = .103). The median increase in performance from t1 to t6 was 0.38, around five times higher than from p1 to p2 (0.08). For blocks t1 to t2 we also found a non-significant (*p* = .026) median increase of 0.08, comparable to the probe block performance change.

To directly test whether the change in performance from p1 to p2 was different from that observed from t1 to t2, we computed a two-sided bootstrap hypothesis test on the between-block differences in proportion of trials solved. We found no statistically significant difference in performance change between the two pairs of blocks (*p* = .568).

To assess how well training amount and working memory capacity can account for task performance, we ran binomial regressions using main effects of block number, participant working memory score, and each of the three per-block strategy report values (reported proportion of trials using “intuition”, “work-it-out”, and “try-and-see” strategies). We also included interaction terms for block number and each of the three strategies. These regressions attempt to predict the number of trials successfully solved by participants across both groups. For the training blocks, we found a significant positive association between block number and performance (*β* = 0.236, 95% CI [0.203, 0.269], *p* < .001). We also found a significant negative effects of use of the “try-and-see” strategy (*β* = −1.379, 95% CI [−1.938, −0.818], *p* < .001) and use of the “intuition” strategy (*β* = −0.400, 95% CI [−0.785, −0.016], *p* < .001). No other main effects or interaction effects were statistically significant (*p* > .100; Full results in Supplementary Table S1).

For the probe blocks, block number (*β* = 0.492, 95% CI [0.268, 0.715], *p* < .001) and “try-and-see” strategy (*β* = −1.195, 95% CI [−1.843, −0.548], *p* < .001) had a significant association with success probability, but the other variables did not (Full results in Supplementary Table S2).

These results are consistent with a training-dependent increase in the probability of trial success. The results also suggest that stronger use of strategies involving trial and error or “intuitive” decisions are associated with poorer performance independent of training.

#### Time Between Rule Uses Decreased More for Trained Than Untrained Conditions.

We computed the mean time between successive rule applications a function of block number. We observed a clear downward trend for the trained conditions for both participant groups (Figure 2C).

To determine whether between-rule time differed between the two participant groups, we performed two-sided bootstrap hypothesis tests for each of the probe and training blocks. We found no statistically significant differences in proportion of trials solved for participants in group A compared to group B for any block (*p* > .014, in all blocks; *p* > .043 for blocks p1, p2, t1, and t6). Based on this observation, we pooled data across the two groups for our subsequent analyses of between-rule time.

We computed the differences in median time between rule uses for the last and first training blocks (t6 and t1), the post-training and pre-training probe blocks (p2 and p1), and the first two training blocks (t2 and t1) (Figure 2D). One-sided bootstrap hypothesis tests found significant reductions in mean time between rule uses t1 to t6 and p1 to p2 (*p* < 0.001). There was no significant difference for t1 to t2 (*p* = .012).

To directly test whether the change in median between-rule time from p1 to p2 was different from that observed from t1 to t2, we computed a two-sided bootstrap hypothesis test on the between-block differences in between-rule time (that is p2 − p1 compared to t2 − t1). We found a statistically significant difference in between-rule time between the two pairs of blocks (*p* < .001), with median difference for the probe blocks (−1.70) greater in magnitude than that of the first two training blocks (−0.83).

To further assess the association between training amount, working memory, and median time between rule uses, we ran gamma regressions. Predictors were as in the performance regression described above. For training blocks, we found statistically significant direct effects of block number (*β* = −0.083, 95% CI [−0.102, −0.064], *p* < 0.001), working memory score (*β* = −0.056, 95% CI [−0.096, −0.015], *p* = 0.007), use of the “try-and-see” strategy (*β* = 0.41, 95% CI [0.114, 0.705], *p* = 0.007), and use of the “intuition” strategy (*β* = 0.831, 95% CI [0.616, 1.045], *p* < 0.001). The interaction effect of “intuition” strategy by block was also statistically significant (*β* = −0.097, 95% CI [−0.15, −0.044], *p* < 0.001), indicating that greater use of intuition predicts shorter median time between rule uses as training increases. The other direct effect and interaction effects were not statistically significant (Full results in Supplementary Table S3)

For the probe blocks, we found statistically significant main effects of block number (*β* = −0.369, 95% CI [−0.469, −0.269], *p* < 0.001), use of the “work-it-out” strategy (*β* = 0.48, 95% CI [0.219, 0.74], *p* < 0.001), use of the “try-and-see” strategy (*β* = 0.734, 95% CI [0.484, 0.984], *p* < 0.001), and use of the “intuition” strategy (*β* = 0.481, 95% CI [0.26, 0.703], *p* < 0.001). None of the other main or interaction effects were statistically significant (Full results in Supplementary Table S4).

#### Within-Block Changes in Probe Trial Performance Are Comparable to Those of Training Trials.

To assess whether the small improvements in probe trial performance were attributable to generalisation rather than direct learning on those tasksets, we conducted a regression analysis on participants’ per-trial performance. This allows us to identify changes in performance during individual blocks, rather than changes in block-averaged performance. If performance on the probe taskset improved due to transfer from the training taskset, we would expect performance to increase more rapidly over the two probe blocks than it did over the first two training blocks using that taskset.

We used a hierarchical Bayesian logistic regression model to predict per-trial success using main effects of cumulative trial number (counting across blocks, separately per ruleset), counterbalance group (A or B), and trial type (probe versus training). The model also included interaction terms for counterbalance group by probe condition, counterbalance group by cumulative trial number, and cumulative trial number by probe condition. The model allowed both varying slopes and varying intercepts for each participant. We report the resulting estimates of learning rates and baseline performance levels per group and trial condition. A full summary of means and credible intervals for each fitted parameter of the model, together with corresponding odds ratios, is given in Supplementary Table S5.

Both groups had similar baseline performance (estimated probability of trial success) at the first training trial (Group A: *M* = .160, 95% HDI [0.125, 0.95]; Group B: *M* = .217, 95% HDI [0.167, 0.265]) and the first probe trial (Group A: *M* = .170, 95% HDI [.107, .237]; Group B: *M* = .100, 95% HDI [.049, .155]).

Group A had a positive learning rate on training trials (*β* = 0.032 on logit scale, 95% HDI [0.026, 0.037]; OR = 1.032, 95% HDI [1.026, 1.037]). This corresponds to a mean 3.2% increase in the odds of success for each trial encountered. Group B also had a positive learning rate on training trials (*β* = 0.025 on logit scale, 95% HDI [0.019, 0.037]; OR = 1.025, 95% HDI [1.019, 1.031]), corresponding to a comparable 2.5% per-trial increase in the odds of success. Group B had a slightly slower mean learning rate on training trials than did group A (*β*(B) − *β*(A) = −0.007, 95% HDI [−0.015, 0.001]), but the credible interval for the difference in learning rates between groups spans 0.

For probe trials, rates of performance improvement for Group A (*β* = 0.039, 95% HDI [0.015, 0.063]; OR = 1.040, 95% HDI [1.015, 1.065]) and Group B (*β* = 0.052, 95% HDI [0.021, 0.082]; OR = 1.054, 95% HDI [1.021, 1.086]) were again comparable. The mean difference in slopes was uncertain (*β*(B) − *β*(A) = 0.013, 95% HDI = [−0.021, 0.050]), providing no credible evidence for a difference in learning rates on probe trials.

**Figure 3.**
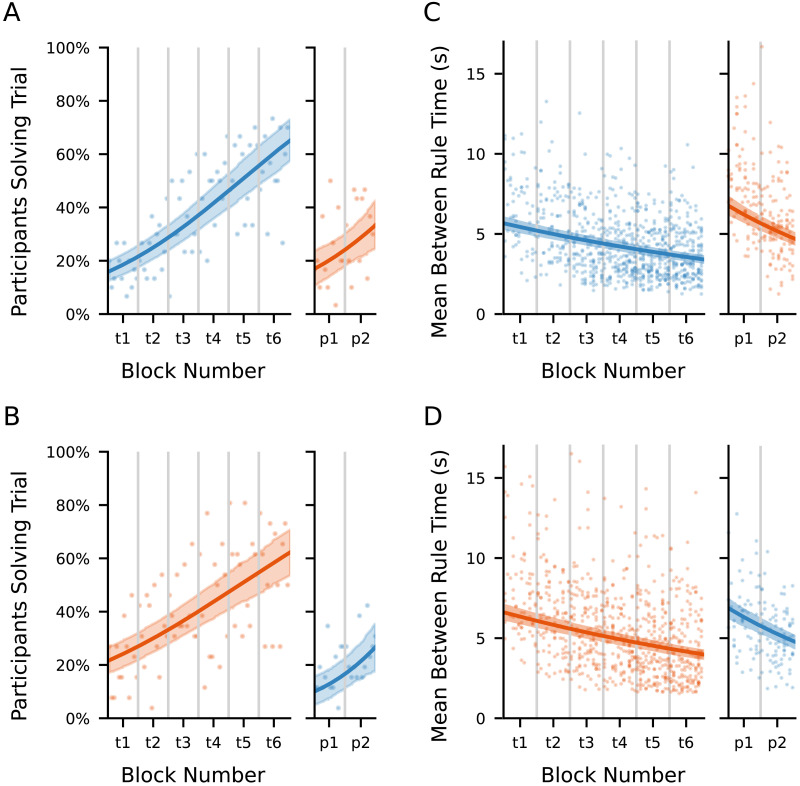
Per-trial trends in performance for consecutive training blocks and non-consecutive probe blocks. (A) Observed proportions of Group A participants solving each consecutive trial (points), and the posterior mean trend line predicting success as a function of trial number. Shaded area is the 95% posterior credible interval around the mean. Left plot shows training trials, right plot shows non-consecutive probe blocks arranged continuously by rank order of trial number. (B) As A, but for participant Group B. (C) Observed means of per-trial per-participant time between rule uses for successful trials by Group A participants (points). The line is the posterior mean trendline produced by the Bayesian regression model predicting mean between rule times as a function of trial number. The shaded area is the 95% posterior credible interval around this mean. Left plot shows training trials, right plot shows probe trials arranged by rank order of trial number. (D) As C, but for participant Group B.

To check for a difference in learning rate between probe and training trials within each group, we computed posterior estimates of the difference between the probe trial learning rates and the training trial learning rates. For Group A, we found no evidence for a difference in probe and training learning rates (*β*(Probe) − *β*(Training) = 0.007, 95% HDI = [−0.017, 0.033]). Similarly, for Group B, we found no evidence for a difference in probe and training learning rates (*β*(Probe) − *β*(Training) = 0.027, 95% HDI = [−0.005, 0.059]). Learning curves for Groups A and B are shown in Figure 3A and B.

These results are consistent with performance improvements due to direct practice on each taskset, providing little credible evidence of transfer of learning from the training to the probe tasksets.

#### Probe Trial Preparation Time Reduced Faster Than Training Trial Preparation Time.

We used a hierarchical Bayesian log-normal regression to examine how participants’ mean between-rule times per trial varied across groups, task conditions, and trial outcomes (success or failure). Details of the model are given in the Methods section. Means and credible intervals for all estimated parameters are given in Supplementary Table S6.

Averaging across participants, preparation time was found to decrease by 0.70% per trial for training trials (95% HDI [−0.79%, −0.62%]; *P*(effect < 0) = 1.000). This compounds to 8.08% per 12-trial block (95% HDI [−9.03%, −7.19%]). In probe trials, the corresponding decrease in preparation time per trial was 1.51% (95% HDI [−1.81%, −1.21%]; *P*(effect < 0) = 1.000). For probe trials, the rate of reduction in preparation time was therefore faster by an additional 0.81% compared to training trials (95% HDI [0.52%, 1.11%]; *P*(effect > 0) = 1.000).

The rate of change in preparation time differed slightly between probe and training contexts, with preparation times decreasing 0.8% more quickly per trial during probe trials than during training trials (95% HDI [−1.1%, −0.5%]; *P*(effect > 0) = 0.00). Together, these results indicate clear group differences across conditions, robust effects of trial success, and small but reliable differences in learning dynamics between probe and training tasks.

In training trials, Group B preparation times were on average 16.6% longer than those of Group A (95% HDI [4.3%, 27.6%]; *P*(effect > 0) = 0.998). In contrast, during probe trials Group B had shorter preparation times, with a mean of 12.7% less than Group A (95% HDI [−19.3%, −5.6%]; *P*(effect > 0) = 0.000). Preparation times in successful trials were also approximately 42.1% lower on average than in failed trials, regardless of group (95% HDI [−43.4%, −40.7%]; *P*(effect > 0) = 0.00). Trends in mean between-rule time for successful trials for Groups A and B are shown in Figure 3C and D.

These results are consistent with a direct taskset-specific practice effect in both probe and training trials, alongside a small acceleration in decision preparation during probe trials that may reflect procedural streamlining or general task familiarity.

#### Participants Learned to Select More Efficient Rule Sequences for the Trained Ruleset.

Improvements in participants’ probability of solving tasks was observed concurrently with a reduction in the average time between rule uses. This pattern of behaviour could arise from a trial-and-error problem-solving strategy. If participants strategically direct their choice of rules to a subset deemed most viable, then rapidly use the rules until a solution is found, performance may increase. To determine if participants indeed used such a strategy, we assessed the proportion of their generated rule-use sequences which were optimal in the sense of using the minimum number of rule uses to achieve the goal.

**Figure 4.**
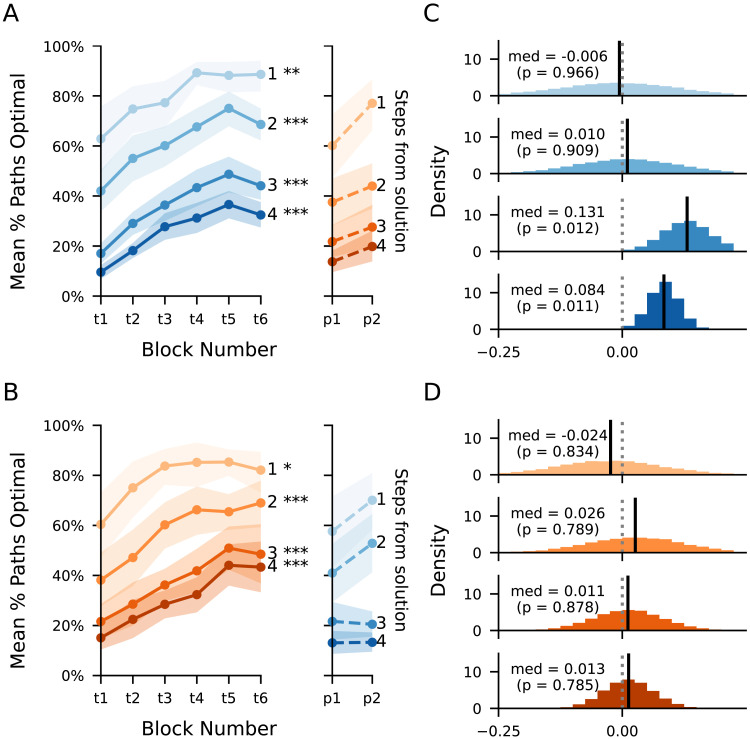
Trends in the proportion of generated rule use sequences which were of optimal length, for successful trials. (A) Mean proportion of participants’ generated action sequences which had optimal (i.e., minimal) length. The start state’s distance from the goal is inset (numbers to right of the lines). Shaded areas are 95% bootstrap confidence intervals for the mean. Plots show data for participant group A (trained with ruleset A and using ruleset B as probe). (B) As in subfigure A, but for participant group B (trained with ruleset B, and using ruleset A as probe). Stars indicate level of significance from bootstrap tests for an increase in percentage optimal trials between the first block and final block (i.e., blocks 2 and 7 for the left training block plots, and blocks 1 and 8 for the right probe block plots). *p* < 0.001 is indicated by “***”, 0.001 < = *p* < 0.01 by “**”, and 0.01 < = *p* < 0.05 by “*”. (C) Bootstrap distributions for the difference (t2 – t1) – (p2 – p1) for ruleset A. This contrast describes the difference in optimality increase during the first training trials and the first probe trials of ruleset A. Inset values are medians of the distribution and p-values from bootstrap hypothesis tests for a difference from zero. Top to bottom plots correspond to distances 1 to 4 from the solution respectively. (D) As in subfigure C, but for ruleset B.

From each state visited by the participants across all successfully solved tasks, we computed the length of the rule sequence used to reach the solution starting from that state. We then computed the percentage of these sequences that had minimum length, according to the pre-computed optimal rule use sequence. Figure 4A and B summarise changes in the percentage of optimal solutions, separating the data by the distance of the start states from the goal state.

For the trained conditions, we observe distinct upward trends in the proportion of solutions that are optimal at distances between one and four steps from the goal. Bootstrap tests for a difference between blocks t1 and t6 find that the increase is statistically significant (*p* < = .001) for distances 2 to 4 in both participant groups. At distance 1, the increase is less robust in the two groups (*p* < = 0.05). For the probe conditions, the upward trend did not reach statistical significance at any distance from the solution in either group.

To compare the change in decision optimality in the first two training blocks using a ruleset to the change in optimality in the two probe blocks using that ruleset, we computed bootstrap distributions for the difference between the former and latter. Figure 4C and D show these distributions for the two rulesets and each of the four distances to the goal. Using bootstrap hypothesis tests for a difference from zero, we observe no statistically significant differences in the change in decision optimality in the first two training and probe trials, except for solution distances 3 and 4 in ruleset A where the test results favour a larger increase in the training condition than the probe condition.

To further distinguish the associations between training, working memory, distance from goal, participant group, and rule sequence optimality, we used binomial regression. We included main effects of block number, working memory score (per block), distance to goal, participant group, and the weighting of each of the three participant-reported strategies (per block). We also included interaction terms for block number by group, block number by working memory score, and each strategy weight by block.

For the training blocks, the main effects of block number (*β* = 0.234, 95% CI [0.134, 0.334], *p* < 0.001), distance to goal (*β* = −0.809, 95% CI [−0.848, −0.769], *p* < 0.001), and use of the “work-it-out” strategy (*β* = 0.751, 95% CI [0.41, 1.093], *p* < 0.001) were all statistically significant. Use of the “try-and-see” strategy (*β* = −0.449, 95% CI [−0.806, −0.093], *p* = 0.013) and “intuition” strategy (*β* = 0.273, 95% CI [0.006, 0.54], *p* = 0.045) did not achieve statistically significant effects at our chosen significance level of 1%. The interaction term for “intuition” strategy use by block number (*β* = 0.093, 95% CI [0.029, 0.157], *p* = 0.004) was also significant, suggesting that stronger use of this strategy was more associated with greater probability of decision optimality in later learning. No other predictors were significant (Full results in Supplementary Table S7).

For the probe blocks, distance to goal (*β* = −0.879, 95% CI [−0.957, −0.801], *p* < 0.001), use of “intuition” (*β* = 0.546, 95% CI [0.214, 0.879], *p* = 0.001), use of “try-and-see” (*β* = −0.588, 95% CI [−1.009, −0.168], *p* = 0.006), and use of “work-it-out” (*β* = 0.754, 95% CI [0.343, 1.166], *p* < 0.001) had significant association with optimality. No other predictors were significant (Full results in Supplementary Table S8).

These results show that the probability of making optimal decisions increased with training at similar rates for both participant groups. Use of both the “intuition” strategy and the “work-it-out” strategy were associated with higher probability of optimal decisions, while use of the “try-and-see” strategy was associated with lower probability of optimal decisions, at least in the probe trials.

### Possible Sources of Transfer

#### Transfer Is Theoretically Possible Using a State-Value Heuristic.

As discussed above, transfer of performance between the trained and untrained conditions should be possible if learning is achieved by improving the accuracy of a function which assigns values to state and task goal pairs. We refer to this function as a state-value heuristic, to emphasise its role as a component of heuristic search (Salhi, 2017) and its role in assigning value estimates to task states.

To demonstrate that transfer could be achieved by reusing a state-value heuristic across tasksets, we devised a parametric state-value heuristic and used it to compute the value of a subset of the states which could be reached in the training or probe tasks. We fitted the state-value heuristic to states from training tasks, and assessed its accuracy in predicting the ground truth value of both held-out states from the training tasks and all other states from the probe tasks.

The model's median accuracy on test states from ruleset A after fitting with states from ruleset A was 0.815 (Q1 = 0.785, Q3 = 0.876). The corresponding median generalisation accuracy to ruleset B was 0.814 (Q1 = 0.812, Q3 = 0.816). We found comparable results when the training and generalisation groups were reversed (training median = 0.830, Q1 = 0.771, Q3 = 0.852; generalisation median = 0.820, Q1 = 0.818, Q3 = 0.821). Reported quartiles are computed from 30-fold cross-validation model fits.

We note that, of the 14 fitted parameters, only those corresponding to the difference in lengths between the state and goal strings, the length of the longest common substring between the two, and the length of the current state string were statistically significant at the 1% level in all cross-validation folds. The other parameters were not significant in any fold, for either ordering of training and generalisation datasets.

These results demonstrate that there exist state-value heuristic functions capable of achieving similarly high accuracy on both tasksets used in this study, and that a model fitted to one taskset can generalise to unseen states from the other. Given class-balanced labels (chance = 0.50), accuracies around 0.81 indicate substantial cross-task generalisation under this representation. This suggests that transfer between the two tasksets may be possible if a common state-value heuristic is learned and reused across both. We note, however, that this demonstration is not sufficient to show that all such state-value heuristics would result in transfer. We briefly describe this limitation in the Discussion section.

To assess whether goal and state information were each required to achieve good accuracy in state-value estimation, we repeated the preceding analysis using limited sets of predictors. For a model trained using only the subset of four features exclusively including information about the state (rather than the goal), the median accuracy on test states from ruleset A after fitting with states from ruleset A was 0.698 (Q1 = 0.619, Q3 = 0.741). The corresponding median generalisation accuracy to ruleset B was 0.640 (Q1 = 0.627, Q3 = 0.640). For a model trained using only the subset of four features exclusively including information about the goal (rather than the current state), the median accuracy on test states from ruleset A after fitting with states from ruleset A was 0.472 (Q1 = 0.346, Q3 = 0.657). The corresponding median generalisation accuracy to ruleset B was 0.511 (Q1 = 0.511, Q3 = 0.511).

These results suggest that greater than chance accuracy can be achieved by this model when using only state information, but not when using only goal information. In both cases, accuracy is reduced compared to the model including information about both state and goal. We discuss possible limits to the generality of this finding in the Discussion section.

#### Participants’ Average Cursor Movement Speed Did Not Change Across Blocks.

**Figure 5.**
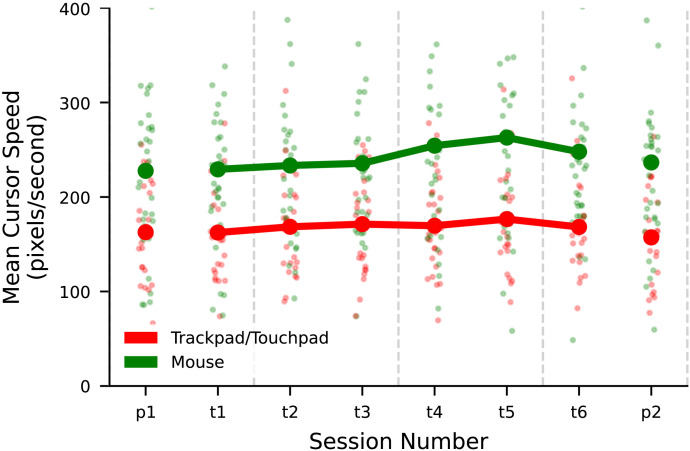
Mean cursor movement speed per block. Pale points are participant per-block means. Heavy points are means of participant means per block, grouped by input device. Movements were assumed to be straight lines between the cursor coordinate where a rule was clicked and the cursor position where the state string was clicked to apply that rule (regardless of the order in which the two were clicked).

To assess whether the observed reduction in time between rule uses on the probe blocks could have been partly attributable to increases in movement speed or accuracy, we computed participants’ average cursor movement speeds in each block. Figure 5 shows trends in mean cursor movement speed during rule use events across blocks.

We used linear regressions to assess trends in cursor movement speed across the training blocks and between the two probe blocks. We used block number and input device as the predictors of cursor movement speed.

For the training blocks, use of a mouse was associated with an increase in mean movement speed of 74.1 pixels per second (95% CI [56.7, 91.5], *p* < 0.001) compared to use of a touchpad. Block number had no statistically significant association with movement speed (*β* = 3.901, 95% CI [−1.144, 8.950], *p* = .129).

Similarly, for the probe blocks, use of a mouse was associated with an increase in mean movement speed of 72.1 pixels per second (95% CI [41.2, 103.0], *p* < 0.001) compared to use of a touchpad. The post-training block was not associated with a statistically significant change in cursor speed compared to the pre-training block (*β* = 3.004, 95% CI [−27.381, 33.388], *p* = .845). Full regression results are given in Supplementary Tables S9 and S10.

## DISCUSSION

The present study sought to determine if improvements in performance on one transformation problem-solving task could transfer to another identically presented task which did not share the same transformation rules. We argued that, while different models of problem-solving can achieve similar performance, they differ in the extent of transfer that they predict. If performance is largely determined by the presence of an accurate state-value heuristic which guides a heuristic search process, then improvements in performance may transfer to other tasks for which the same state-value heuristic can be reused. If performance mainly stems from improvements in pattern recognition and the formation of associations between recognised patterns and contextually appropriate actions, then transfer should be limited to tasks where these associations can be reused. Our tasksets were designed to allow reuse of state-value heuristics, but to prevent reuse of pattern-action associations.

For the trained tasksets, we observed improvements in the proportion of trials correctly solved, the time between successive rule uses, and the average efficiency of the generated rule sequences. We demonstrated that, in the two arbitrary tasksets that we used for training and probe blocks, there existed a state-value heuristic which could be reused across tasksets to achieve transfer. Despite this, we observed only small improvements in performance on the probe trials. These improvements were not statistically distinguishable from those expected due to direct learning during the probe trials. The same pattern was observed regardless of which taskset was used for training and which was used for probe trials. These results are consistent with an absence of transfer between the trained and probe tasks.

It is important to note that the absence of a clear transfer effect is not sufficient to disprove the involvement of state-value heuristics in participants’ problem-solving processes. Several other explanations, including interference effects, changes in problem-solving mechanisms due to learning, and simple failure to reuse the same mechanisms across tasks, can all account for the same result. We now discuss the extent to which each of these explanations is consistent with our results.

### Possible Reasons for the Absence of Transfer Between Tasksets

#### Variations in Working Memory Load.

Switching tasks to an unfamiliar ruleset could lead to a reduction in performance by increasing participants’ working memory load. In our experiment, this may have arisen in at least two ways: from the need retain the new rules in memory in order to choose which ones to use; and from the need to actively disregard any automatically recalled associations between the task state and the former and now unavailable rules. This additional working memory load may hamper task performance if the mechanisms supporting task performance are contingent upon working memory (Sweller, 1988).

Existing literature suggests that novice problem-solvers depend more upon working memory-heavy problem-solving processes than do experts in that task domain (Gick, 1986; Gobet, 1998; Gobet & Charness, 2018; Gobet & Waters, 2023). As such, we expected to observe a positive association between working memory score and performance, and that this association would reduce as training progressed. In fact, we did not observe a statistically significant association between working memory score and performance, time between actions, or solution efficiency when participants’ self-reported strategies were controlled for.

This limits our ability to assess whether performance on the probe trials was impeded by excess working memory load, as our participants’ performance was insensitive to their natural variations in working memory capacity. A future study could better distinguish the influence of working memory load by directly affecting participants available working memory capacity, such as by introducing a concurrent distractor task alongside the string transformation task. By varying the additional working memory load and assessing how it affects performance, participants’ remaining working memory capacity during each block could be assessed.

#### Changes in Problem-Solving Method.

Our analyses suggested that participants did not on average change their strategy in a consistent way across sessions. While individuals’ strategy reports often changed between blocks, interaction effects between strategy and block were not statistically significant predictors of performance, solution efficiency, or time between rule uses. This suggest that, if participants’ method of problem-solving changed with training, the changes were not consistent with a switch from one of the pre-defined strategies to another, or the participants were unaware of their change of method.

Our analysis of participants’ solution efficiency provided evidence of changes in problem-solving behaviour due to training. Participants tended to choose more efficient action sequences in the latter stages of training, as demonstrated by the increased frequency with which they generated minimal-length rule use sequences. This increase in efficiency was observed in the training taskset for start states at distances of up to four steps from the goal. In the probe taskset, there was no significant increase in efficiency at any distance from the goal for either participant group. Disregarding the potential effect of cognitive load described above, this contrast suggests that the dominant problem-solving method used in the trained taskset was either reused for the probe taskset, where it happened to be ineffective, or that the methods used in the two cases differed.

It should also be noted that the algorithm used to achieve better problem-solving performance in the later trials of the trained condition is not the only possible source of transfer. Some explicit strategies, such as avoiding making changes to parts of the task state already matching the goal, can be effective in a broad range of tasks, independent of the ruleset available in those tasks. Reuse of these strategies could lead to improved performance on the probe condition. While we did not observe performance improvements consistent with transfer on the probe condition, the reduction in between-rule time was larger in the probe condition than the equivalent trials of the trained condition. This suggests that participants made faster decisions after training, even on tasks using a relatively less practiced ruleset. It is plausible that this is a consequence participants’ having established a higher-level strategy enabling them to spend less time considering which actions to take. However, it is also plausible that participants were simply hurrying to complete the final probe block due to loss of interest after four days of the study. We suggest that future studies should attempt to distinguish the effect of explicit strategy and more implicit learning processes to better determine how they affect transfer of performance, decision time, and action optimality.

#### Failure to Re-Use the State-Value Heuristic.

The observed absence of transfer between tasksets could have occurred if a state-value heuristic was used in the trained taskset, but was not reused in the probe taskset. It is plausible that different task domains could trigger learners to acquire specific taskset-dependent state-value heuristics. Ensuring independence between the computational mechanisms underlying skilled performance in different tasks would have the advantage of reducing learning-related interference between the tasks. This form of interference, different from the working memory-related interference described above, is closely related to the stability-plasticity dilemma (French, 1999; McCloskey & Cohen, 1989; Mermillod et al., 2013). By separating learning by taskset, changes in neural tuning brought about by practicing one taskset can be prevented from diminishing performance on another taskset. However, the same independence would also prevent beneficial transfer or generalisation across tasks (Gabriel et al., 2024). Although there was no generalisation across tasksets in our study, there was generalisation within tasksets, as demonstrated by the gradual improvement in performance over the training trials. It is unclear why, if a state-value function was responsible for this generalisation, it could not also allow generalisation across tasksets.

We note that the pattern-recognition and association account of problem-solving skill can also protect against the plasticity-related form of interference, as the associations between patterns and actions are specific to the available transformation rules. It is unclear whether the use of distinct transformation rules for the two tasksets caused participants to favour the pattern-recognition and association mechanism of problem-solving, or whether it caused them to learn distinct state-value functions for the two tasksets. Further research will be required to determine more specific conditions under which cross-taskset generalisation can occur, and the mechanisms through which it arises.

#### Limitations of the State-Value Transfer Demonstration.

Our analysis using an example state-value heuristic was intended to show that there exist heuristics for which transfer can be achieved through reuse across tasks. This analysis was not intended to demonstrate that transfer is possible using any such heuristic. It is possible that a failure of transfer could occur even when a state-value heuristic was used, if that heuristic happened to achieve better performance on the trained rather than the untrained task. While we cannot discount this possibility, we argue that it is unlikely to be the case for the following reasons.

We fitted our example state-value heuristic to states observable in the training taskset using 30-fold cross-validation. This method allowed us to assess the ability of the model to approximate our binary ground-truth state-value. We found that the model achieved around 80% accuracy in both testing and generalisation. We also found that our example state-value heuristic achieved poorer accuracy when limited to using goal-only or state-only features. Although we did not demonstrate that this would be true for all heuristics and ground truth value assignments, we reason that similar effects would be found is most cases. This is because the value of a state should always be related to whether the task can be solved from that state, and this possibility is not determined by either the state or the goal alone, but by the combination of the two. Indeed, a given state may be on a solution path for one goal, but a dead-end for another. Consequently, using only information about the current state could allow some low-value states to be identified (for example, if they were too short to be on any solution path), but using only goal information would not be sufficient to assign an accurate value to a state.

We also observed that the only statistically significant predictors of state-value were the difference between the length of the state and goal strings, the length of the longest common subsequence between the two, and the length of the state string. As it is possible to achieve both high accuracy on both the training and generalisation states using only these features, and as none of these features is taskset-specific, we conclude that it should be possible for a broad range of state-value heuristics to achieve transfer.

### Implications for Training of Problem-Solving Skills

The findings of this study may have practical implications for education in areas such as mathematical and computational problem-solving. Specifically, the observed failure of transfer between two sets of tasks with identical presentation but different rulesets suggests that the utility of general problem-solving training may be limited. General training is likely to result in only modest increases in performance on different domain-specific problem-solving skills. However, our results also reiterate that transfer is possible (and indeed necessary for performance improvements) between tasks which reuse the same ruleset. Drawing inspiration from the literature on transfer in perceptual learning (Carvalho & Goldstone, 2019), future research on problem-solving may investigate how to maximise this form of transfer, by appropriately sequencing or selecting training tasks.

### Conclusion

The present study demonstrated that transfer of learning is not guaranteed to occur between transformation tasks with identical visual presentation but differing transformation rules. We argued that, under accounts in which a reusable state-evaluation heuristic should generalise across tasksets, this lack of behavioural generalisation is more consistent with a pattern-recognition and association account than with a heuristic search account, while also noting that absence of transfer does not rule out a role for search in either novel or more practised tasks. We suggest that the general transformation task used in this work could support further studies designed to clarify the conditions under which learning transfers between tasks, or the conditions under which different mechanisms of problem-solving are used. Given the limited transfer observed in this work, we argue that attempts at deriving educational benefit from transfer between task domains are unlikely to be effective when those domains rely upon different transformation rules.

## FUNDING INFORMATION

GG was supported by a Leverhulme Trust Early Career Fellowship. FM was supported in part by a UK Research and Innovation Biotechnology and Biological Sciences Research Council award (BB/X008428/1) and the National Institute for Health and Care Research (NIHR) Leeds Biomedical Research Centre (BRC) (NIHR203331). The views expressed are those of the authors and not necessarily those of the NHS, the NIHR or the Department of Health and Social Care.

## AUTHOR CONTRIBUTIONS

GG conceptualised the experiments, conducted data collection, performed data analysis, drafted the manuscript, and acquired funding. FM assisted with conceptualisation and revision of the manuscript.

## CODE AVAILABILITY STATEMENT

Full analysis scripts for all outcomes reported in this paper are available via the OSF repository (osf.io/mb94g).

## ETHICS APPROVAL

Ethical approval was granted by the University of Leeds School of Psychology ethical review board (Reference: PSYC-798).

## CONSENT TO PARTICIPATE

All participants provided informed consent to participate in the study.

## CONSENT FOR PUBLICATION

All participants provided informed consent allowing publication of their anonymised data and analyses resulting therefrom.

## Supplementary Material


