# Supplementary material for: No Evidence of Transfer of Learning Between Problem-Solving Tasks Using Different Transformation Rules
Source: Open Mind (Camb). 2026 Mar 15;10:261–86. doi: 10.1162/OPMI.a.338 (PMC13056334; doi:10.1162/OPMI.a.338)
Supplement: Supplementary file 1 [file opmi-10-261-s001.pdf]

Supplementary Materials

This document contains supplementary figures, methods, and tables for the paper "No evidence of transfer of learning between problem-solving tasks using different transformation rules". Data and analysis scripts are available from the corresponding OSF repository (osf.io/mb94g).

Supplementary Figures

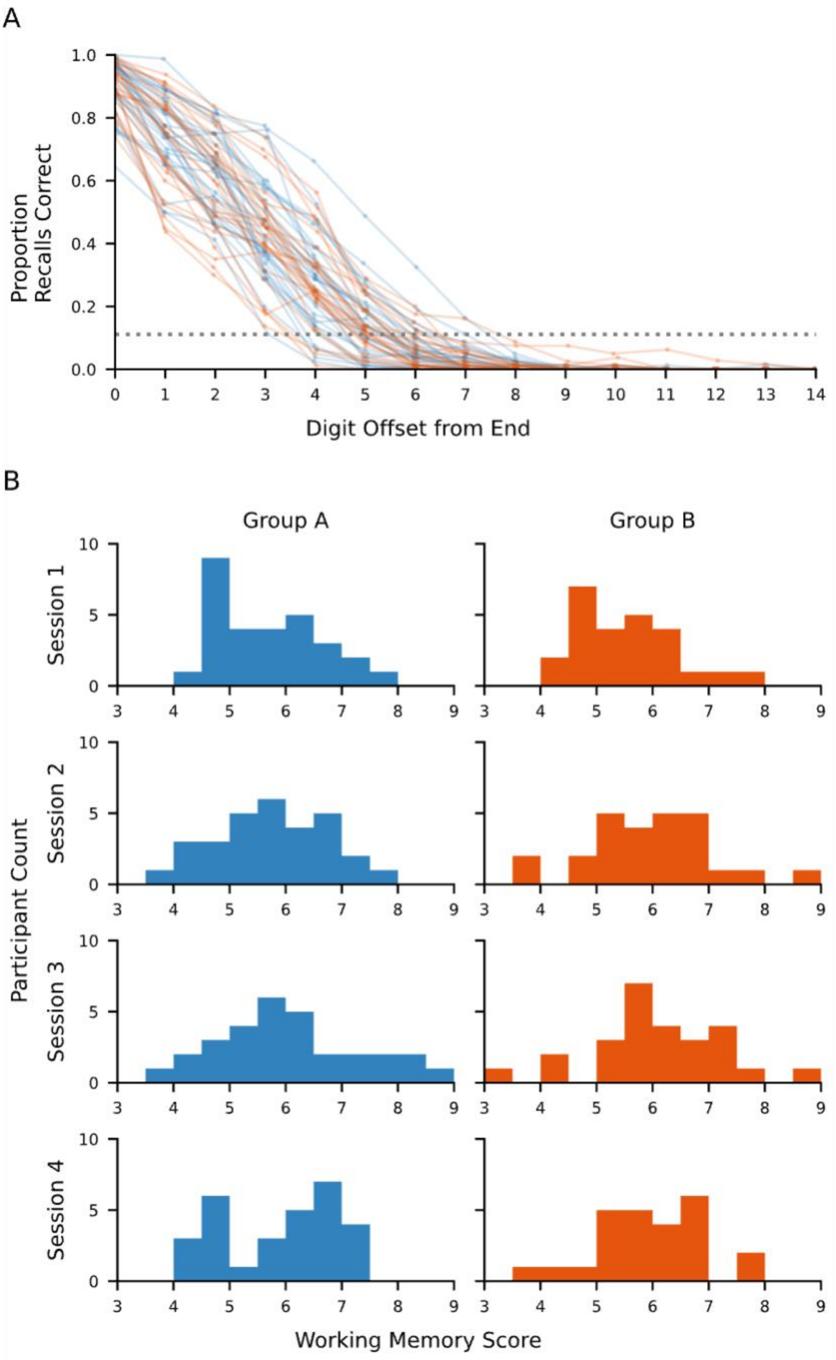

Figure 1 *– Distributions of participant working memory scores across sessions. (A) Trends in proportion of correctly recalled digits as a function of their distance from the end of the digit string. Horizontal dotted line represents chance accuracy. Blue lines are for participants in group A; orange for group B. (B) Histograms of participant working memory scores across sessions.*

Supplementary Methods

Specification of Per-Trial Preparation Time Model

We used a hierarchical Bayesian regression model implemented in PyMC to analyse the mean time between rule uses per trial. Because preparation times are strictly positive and positively skewed, we modelled them on the log scale using a log-normal likelihood. The model estimated both population-level (fixed) effects and participant-level (random) effects, allowing us to quantify overall group differences while accounting for individual variability in baseline between-rule time, rate of change of between-rule time, and relative differences in between-rule time on probe and training trials.

To sample the posterior, we used four chains of 2000 post-warmup samples (2000 warmup iterations) for a total of 8000 posterior draws. We assessed convergence using the Gelman–Rubin statistic ($\hat{R}$), with all parameters having $\hat{R} < 1.01$.

Model Parameters

The following parameters are used in the model:

- T_{ip} represents the mean between-rule time for trial i and participant p.
- $group_{ip}$ is an indicator variable with value 0 if participant p was in group A, and value 1 if they were in group B.
- $success_{ip}$ is an indicator variable with value 0 if the trial was unsuccessful and 1 if it was successful.
- $probe_{ip}$ is an indicator variable with value 0 for training trials and 1 for probe trials
- $trial_{ip}$ is a cumulative trial number, counting trials of tasks from ruleset A separately from trials of tasks from ruleset B.
- u_p are participant-specific parameters allowing varying slopes or intercepts.

Model Specification

The logarithm of the mean between-rule time per participant and trial is specified by

$$log(T_{ip}) \sim \ Normal(\mu_{ip}, \sigma),$$

where the standard deviation parameter follows an exponential distribution

$$\sigma \sim Exponential(1),$$

and the mean contains the main effect, interaction effects, and random slope and intercept terms per participant

$$\mu_{ip} = \alpha_p + \beta_{group} \cdot group_{ip} + \beta_{success} \cdot success_{ip} + u_p^{trial} \cdot trial_{ip} * u_p^{probe} \cdot probe_{ip} + \beta_{\{group \times probe\}} \cdot (group_{ip} \times probe_{ip}) + \beta_{\{trial \times probe\}} \cdot (trial_{ip} \times probe_{ip}).$$

All beta coefficients used weakly regularising priors:

$$\beta \sim \mathrm{Normal}(0, 5)$$

Priors for the per-participant intercept, slope, and probe effect were given by a multivariate normal distribution with covariance matrix constructed using an LKJ prior.

$$\begin{pmatrix} \alpha_p \\ u_p^{\mathrm{trial}} \\ u_p^{\mathrm{probe}} \end{pmatrix} \sim MultivariateNormal\left(\begin{pmatrix} \mu^{(\alpha)} \\ \mu^{(trial)} \\ \mu^{(probe)} \end{pmatrix}, \Sigma \right)$$

$$\mu^{(\alpha)}, \mu^{(trial)}, \mu^{(probe)} \sim Normal(0, 5)$$

$$\mathrm{R} \sim \mathrm{LKJ}(3, \eta = 2)$$

$$\Sigma = \begin{pmatrix} \sigma_1 & 0 & 0 \\ 0 & \sigma_2 & 0 \\ 0 & 0 & \sigma_3 \end{pmatrix} R \begin{pmatrix} \sigma_1 & 0 & 0 \\ 0 & \sigma_2 & 0 \\ 0 & 0 & \sigma_3 \end{pmatrix}$$

$$\sigma_j \sim Exponential(1), \qquad j \in \{1, 2, 3\}$$

Exporting of Bayesian Model Parameters (Preparation Time)

We exported a complete set of summary statistics fort the per-trial model of mean preparation time, including all population-level parameters, random-effect variances, and random-effect correlations. Summaries were exported as a single CSV file (`bayesian_model_summary.csv`). The included parameters and summary statistics are listed below.

Fixed-effect parameters

- beta_group (group A vs. B)
- beta_success (successful vs. failed trial)
- beta_group_probe (group × probe interaction)
- beta_trial_probe (trial × probe interaction)
- mu_intercept, mu_trial_slope, mu_probe_effect (population means of random-effect components)

For each population-level regression coefficient, we report:

- mean posterior estimate
- standard deviation
- posterior median
- 95% highest density interval (HDI)

Random-effect variance components

Standard deviations of participant-level variability for:

- intercepts
- trial slopes
- probe effects (intercept and slope)

These quantify the degree of heterogeneity across participants in baseline performance, learning rate, and condition-specific responsiveness.

Specification of Per-Trial Success Probability Model

We used a hierarchical Bayesian regression model implemented in PyMC to analyse the mean time between rule uses per trial. The model's structure was similar to that used by the preparation time model, and the training process was identical.

Model Parameters

The following parameters are used in the model:

- P_{ip} represents the probability that trial i for participant p was solved.
- $group_{ip}$ is an indicator variable with value 0 if participant p was in group A, and value 1 if they were in group B.
- $probe_{ip}$ is an indicator variable with value 0 for training trials and 1 for probe trials
- $trial_{ip}$ is a cumulative trial number, counting trials of tasks from ruleset A separately from trials of tasks from ruleset B.
- u_p are participant-specific parameters allowing varying slopes or intercepts.

Model Specification

$$logit(P_{ip}) = \alpha_p + \beta_{group} \cdot group_{ip} + u_p^{trial} \cdot trial_{ip} + u_p^{probe} \cdot probe_{ip} + u_p^{\{trial \times probe\}} \cdot trial_{ip} \cdot probe_{ip} + \beta_{\{group \times probe\}} \cdot (group_{ip} \times probe_{ip}) + \beta_{\{trial \times group\}} \cdot (trial_{ip} \times group_{ip}) + \beta_{\{trial \times probe\}} \cdot (trial_{ip} \times probe_{ip}) + \beta_{\{trial \times group \times probe\}} \cdot (trial_{ip} \times group_{ip} \times probe_{ip}).$$

All beta coefficients used the same priors:

$$\beta \sim \mathrm{Normal}(0, 1)$$

Priors for the per-participant intercept, slope, and probe effect were given by a multivariate normal distribution with covariance matrix constructed using an LKJ prior.

$$\begin{pmatrix} \alpha_p \\ u_p^{\text{trial}} \\ u_p^{\text{probe}} \\ u_p^{\{\text{trial} \times \text{probe}\}} \end{pmatrix} \sim MultivariateNormal(\mu,\ \Sigma)$$

$$\mu \sim Normal(0, 1)$$

$$\mathrm{R} \sim \mathrm{LKJ}(4, \eta = 2)$$

$$\Sigma = \begin{pmatrix} \sigma_1 & \cdots & 0 \\ \vdots & \ddots & \vdots \\ 0 & \cdots & \sigma_4 \end{pmatrix} R \begin{pmatrix} \sigma_1 & \cdots & 0 \\ \vdots & \ddots & \vdots \\ 0 & \cdots & \sigma_4 \end{pmatrix}$$

$$\sigma_j \sim Exponential(1), \qquad j \in \{1, 2, 3, 4\}$$

Exporting of Bayesian Model Parameters (Success Probability)

We exported a complete set of summary statistics fort the per-trial model of mean preparation time, including all population-level parameters, random-effect variances, and random-effect correlations.

Fixed-effect parameters

- beta_group (group A vs. B)
- beta_group_probe (group × probe interaction)
- beta_trial_group (trial × group interaction)
- beta_trial_probe (trial × probe interaction)
- beta_trial_group_probe (trial × group × probe interaction)
- mu_intercept, mu_trial_slope, mu_probe_effect, mu_probe_slope (population means of random-effect components)

Random-effect variance components

Standard deviations of participant-level variability for:

- intercepts

- trial slopes
- probe effects (intercept and slope)

Supplementary Tables

Supplementary Table S1

Binomial Regression: N Successes in Training Blocks

Predictor	B	SE	z	p	95% CI Lower	95% CI Upper	exp(coef)
const	-1.470	0.174	-8.456	< .001	-1.811	-1.130	0.230
Block Number	0.236	0.017	14.093	< .001	0.203	0.269	1.266
WM Score	0.057	0.035	1.624	0.104	-0.012	0.126	1.059
Try and See	-1.378	0.286	-4.825	< .001	-1.938	-0.818	0.252
Work it Out	0.308	0.273	1.131	0.258	-0.226	0.842	1.361
Intuition	-0.400	0.196	-2.040	0.041	-0.785	-0.016	0.670
Try-and-see x Block	0.076	0.068	1.118	0.264	-0.057	0.209	1.079
Work-it-Out x Block	0.089	0.065	1.372	0.170	-0.038	0.215	1.093
Intuition x Block	0.071	0.047	1.507	0.132	-0.021	0.164	1.074

Supplementary Table S2

Binomial Regression: N Successes in Probe Blocks

Predictor	B	SE	z	p	95% CI Lower	95% CI Upper	exp(coef)
const	-1.835	0.322	-5.705	< .001	-2.466	-1.205	0.160
Block Number	0.492	0.114	4.310	< .001	0.268	0.715	1.635
WM Score	0.165	0.068	2.422	0.015	0.032	0.299	1.180
Try and See	-1.195	0.330	-3.617	< .001	-1.843	-0.548	0.303
Work it Out	-0.063	0.298	-0.212	0.832	-0.647	0.521	0.939
Intuition	-0.577	0.238	-2.425	0.015	-1.043	-0.111	0.562
Try-and-see x Block	-0.211	0.406	-0.521	0.602	-1.007	0.584	0.809
Work-it-Out x Block	0.943	0.451	2.094	0.036	0.060	1.826	2.568
Intuition x Block	-0.240	0.319	-0.751	0.453	-0.866	0.386	0.787

Supplementary Table S3

Gamma Regression: Preparation Time in Training Blocks

Predictor	B	SE	z	p	95% CI Lower	95% CI Upper
const	1.604	0.101	15.950	< .001	1.407	1.802
Block Number	-0.083	0.010	-8.677	< .001	-0.102	-0.064
WM Score	-0.056	0.021	-2.691	0.007	-0.096	-0.015
Try and See	0.410	0.151	2.719	0.007	0.114	0.705
Work it Out	0.364	0.154	2.360	0.018	0.062	0.667
Intuition	0.831	0.109	7.589	< .001	0.616	1.045
Try-and-see x Block	0.062	0.037	1.671	0.095	-0.011	0.136
Work-it-Out x Block	-0.049	0.037	-1.311	0.190	-0.121	0.024
Intuition x Block	-0.097	0.027	-3.581	< .001	-0.150	-0.044

Supplementary Table S4

Gamma Regression: Preparation Time in Probe Blocks

Predictor	B	SE	z	p	95% CI Lower	95% CI Upper
const	1.695	0.152	11.124	< .001	1.396	1.993
Block Number	-0.369	0.051	-7.229	< .001	-0.469	-0.269
WM Score	-0.055	0.033	-1.696	0.090	-0.119	0.009
Try and See	0.734	0.127	5.758	< .001	0.484	0.984
Work it Out	0.480	0.133	3.606	< .001	0.219	0.740
Intuition	0.481	0.113	4.263	< .001	0.260	0.703
Try-and-see x Block	-0.090	0.166	-0.543	0.587	-0.416	0.236
Work-it-Out x Block	-0.285	0.211	-1.350	0.177	-0.698	0.129
Intuition x Block	0.006	0.147	0.043	0.966	-0.282	0.294

Supplementary Table S5

Bayesian Regression: Performance

Predictor	mean	sd	hdi_2.5%	hdi_97.5%	mcse_mean	mcse_sd	ess_bulk	ess_tail	r_hat
beta_group	0.168	0.174	-0.165	0.524	0.004	0.002	1817.000	2528.000	1.000
beta_group_probe	-0.406	0.358	-1.111	0.288	0.005	0.004	4702.000	4986.000	1.000
beta_trial_group	-0.146	0.085	-0.317	0.018	0.001	0.001	4590.000	3913.000	1.000
beta_trial_probe	0.072	0.709	-1.331	1.423	0.008	0.008	7305.000	5353.000	1.000
beta_trial_group_probe	0.278	0.386	-0.445	1.054	0.005	0.004	5513.000	6116.000	1.000
mu_intercept	-0.719	0.117	-0.947	-0.494	0.003	0.001	1997.000	3618.000	1.000
mu_trial_slope	0.671	0.058	0.553	0.778	0.001	0.001	5133.000	4924.000	1.000
mu_probe_effect	0.270	0.254	-0.264	0.730	0.004	0.003	4664.000	5454.000	1.000
mu_probe_slope	0.080	0.709	-1.300	1.480	0.008	0.008	7107.000	5407.000	1.000

Supplementary Table S6

Bayesian Regression: Preparation Time

Predictor	mean	sd	hdi_2.5%	hdi_97.5%	mcse_mean	mcse_sd	ess_bulk	ess_tail	r_hat
beta_group	0.153	0.051	0.051	0.249	0.001	0.001	1486	2760	1
beta_success	-0.547	0.012	-0.570	-0.524	0.000	0.000	15797	6486	1
beta_group_probe	-0.136	0.040	-0.216	-0.060	0.001	0.000	2872	5096	1
beta_trial_probe	-0.008	0.002	-0.011	-0.005	0.000	0.000	3538	4928	1
mu_intercept	2.278	0.037	2.209	2.353	0.001	0.000	2236	4029	1
mu_trial_slope	-0.007	0.000	-0.008	-0.006	0.000	0.000	5059	6112	1
mu_probe_effect	0.177	0.033	0.110	0.240	0.001	0.000	1775	3282	1
sigma_intercept_RE	0.995	0.989	0.000	2.920	0.008	0.018	10219	3991	1
sigma_trial_RE	1.005	0.985	0.000	2.962	0.008	0.017	10429	4660	1
sigma_probe_RE	0.999	1.005	0.000	3.059	0.009	0.019	9935	4034	1
sigma_residual	0.386	0.004	0.379	0.393	0.000	0.000	20091	5877	1

Supplementary Table S7

Binomial Regression: Action Optimality in Training Blocks

Predictor	B	SE	z	p	95% CI Lower	95% CI Upper	exp(coef)
const	0.575	0.214	2.682	0.007	0.155	0.994	1.776
Block Number	0.234	0.051	4.580	< .001	0.134	0.334	1.263
WM Score	0.072	0.047	1.538	0.124	-0.020	0.163	1.074
Distance to Goal	-0.809	0.020	-39.835	< .001	-0.848	-0.769	0.445
Group B Effect	-0.052	0.105	-0.497	0.619	-0.257	0.153	0.949
Block Number x Group (B)	0.049	0.025	1.953	0.051	0.000	0.099	1.051
Block Number x WM Score	-0.016	0.011	-1.423	0.155	-0.037	0.006	0.984
Try and See	-0.449	0.182	-2.471	0.013	-0.806	-0.093	0.638
Work it Out	0.751	0.174	4.310	< .001	0.410	1.093	2.119
Intuition	0.273	0.136	2.003	0.045	0.006	0.540	1.314
Try-and-see x Block	0.077	0.045	1.730	0.084	-0.010	0.164	1.080
Work-it-Out x Block	0.063	0.041	1.539	0.124	-0.017	0.144	1.066
Intuition x Block	0.093	0.032	2.868	0.004	0.029	0.157	1.098

Supplementary Table S8

Binomial Regression: Action Optimality in Probe Blocks

Predictor	B	SE	z	p	95% CI Lower	95% CI Upper	exp(coef)
const	0.712	0.259	2.754	0.006	0.205	1.219	2.038
Block Number	0.402	0.336	1.198	0.231	-0.256	1.061	1.495
WM Score	0.076	0.057	1.338	0.181	-0.035	0.186	1.079
Distance to Goal	-0.879	0.040	-22.007	< .001	-0.957	-0.801	0.415
Group B Effect	-0.109	0.134	-0.816	0.415	-0.372	0.153	0.896
Block Number x Group (B)	-0.073	0.173	-0.424	0.671	-0.412	0.266	0.929
Block Number x WM Score	-0.007	0.075	-0.092	0.927	-0.153	0.139	0.993
Try and See	-0.588	0.215	-2.742	0.006	-1.009	-0.168	0.555
Work it Out	0.754	0.210	3.591	< .001	0.343	1.166	2.126
Intuition	0.546	0.170	3.222	0.001	0.214	0.879	1.727
Try-and-see x Block	0.137	0.276	0.496	0.620	-0.404	0.677	1.147
Work-it-Out x Block	0.563	0.305	1.845	0.065	-0.035	1.160	1.755
Intuition x Block	-0.297	0.227	-1.310	0.190	-0.741	0.147	0.743

Supplementary Table S9

Linear Regression: Cursor Movement Speed in Training Blocks

Predictor	B	SE	t	p	95% CI Lower	95% CI Upper
const	155.819	11.229	13.877	< .001	133.731	177.906
Block Number	3.903	2.566	1.521	0.129	-1.144	8.950
Input Device	74.122	8.840	8.385	< .001	56.734	91.511

Supplementary Table S10

Linear Regression: Cursor Movement Speed in Probe Blocks

Predictor	B	SE	t	p	95% CI Lower	95% CI Upper
const	158.502	14.206	11.157	< .001	130.345	186.658
Block Number	3.004	15.331	0.196	0.845	-27.381	33.388
Input Device	72.082	15.581	4.626	< .001	41.201	102.963

Notes on Bayesian Supplementary Tables (S5 and S6)

- HDI (Highest Density Interval): Narrowest interval containing 95% of the posterior mass.
- ESS (effective sample size): Approximate number of independent draws.
- R-hat (Gelman–Rubin statistic): Convergence diagnostic.
- MCSE (mcse_mean, mcse_sd): Monte Carlo standard error for posterior mean and SD.
